# Real time observation of binder jetting printing process using high-speed X-ray imaging

**DOI:** 10.1038/s41598-019-38862-7

**Published:** 2019-02-21

**Authors:** Niranjan D. Parab, John E. Barnes, Cang Zhao, Ross W. Cunningham, Kamel Fezzaa, Anthony D. Rollett, Tao Sun

**Affiliations:** 1 X-ray Science Division, Advanced Photon Source, Argonne National Laboratory, Argonne, IL 60439 USA; 2 The Barnes Group Advisors, Pittsburgh, PA 15143 USA; 3 Department of Materials Science and Engineering, Carnegie Mellon University, Pittsburgh, PA 15213 USA

## Abstract

A high-speed synchrotron X-ray imaging technique was used to investigate the binder jetting additive manufacturing (AM) process. A commercial binder jetting printer with droplet-on-demand ink-jet print-head was used to print single lines on powder beds. The printing process was recorded in real time using high-speed X-ray imaging. The ink-jet droplets showed distinct elongated shape with spherical head, long tail, and three to five trailing satellite droplets. Significant drift was observed between the impact points of main droplet and satellite droplets. The impact of the droplet on the powder bed caused movement and ejection of the powder particles. The depth of disturbance in the powder bed from movement and ejection was defined as interaction depth, which is found to be dependent on the size, shape, and material of the powder particles. For smaller powder particles (diameter less than 10 *μ*m), three consecutive binder droplets were observed to coalesce to form large agglomerates. The observations reported here will facilitate the understanding of underlying physics that govern the binder jetting processes, which will then help in improving the quality of parts manufactured using this AM process.

## Introduction

Additive Manufacturing (AM) is a disruptive technology that adds material in a layer-wise fashion to build complex parts. The layer-by-layer approach offers various advantages over conventional manufacturing which include ability to manufacture complex parts, design flexibility, decreased lead time, prototyping, customized articles, reduced inventory of spares, and on-demand manufacturing^1^. The material choices for AM are also nearly infinite depending on the process chosen. With these advantages, use of AM is growing rapidly in fields of medical, aerospace, automobile, and defense industries^2,3^. ASTM F42 recognizes 7 categories of AM^4^ and this paper focuses on binder jetting AM specifically.

Binder jetting AM uses iterative ink-jet printing of binder material on powder beds to create parts^4,5^. In a typical process, a layer of powder is spread to a desired thickness and the binder is precisely deposited on the powder bed, applying adherent liquid that binds the powder particles together locally. This process is repeated, layer by layer, creating a series of 2D cross-sections. The bound part undergoes curing at a low temperature to produce a “green” part, which can be directly used as sand molds, sintered to form a component, or infiltrated with a third material to produce a composite. Fusion AM processes such as selective laser melting or photo-polymerization are predominantly used for certain materials (metals and polymers for laser/electron beam and photo-polymerization respectively), though some examples of ceramic materials manufactured using fusion processes are present in literature^6,7^. Binder jetting has been used to manufacture metallic parts from stainless steels^8–14^ and other iron alloys^15^, copper^16^, and nickel superalloys^17–19^. Meanwhile, binder jetting for ceramic parts has also been demonstrated for *Al*_2_*O*_3_^20–22^, bioactive glass with hydroxyapatite^23^, barium titanate^24^, hench glass with tricalcium phosphate^25^, plaster of paris^26^, tungsten carbide with 12% Co^27^, and silica sand molds^28^. Binder jetting process exhibits many other advantages. In laser and electron beam metal AM processes, the large heating and cooling rates (typically on the order of 10^3^–10^6^ K/s^29^) often lead to deleterious residual stresses and undesirable microstructures^1^. Since binder jetting process decouples the printing process and subsequent densification post-processing step (typically sintering), thermal residual stresses and highly anisotropic microstructures can be avoided^14,17,28,30–32^. Like all powder bed AM techniques, binder jetting uses the surrounding powder for supporting subsequent layers and hence does not require support structures for building complex parts^10,11,16^. By adding multiple print-heads and nozzles, binder jetting can be readily scaled for printing large parts at accelerated pace^5,16,33^. Further, functionally graded parts can be manufactured by varying the composition of each layer^34^.

Several physical phenomena govern the quality of binder jetted parts, including droplet formation in nozzles, powder flow and packing in powder bed, powder-binder interactions, binder curing, and thermal sintering of the green part^32^. Process parameters that control these processes can be grouped into four distinct categories: 1. powder characteristics (material, shape, particle size distributions), 2. binder characteristics (binder properties, droplet volume, droplet speed, separation between droplets, saturation), 3. part characteristics (nominal dimensions, orientation, location in the print bed, geometric features), and 4. post-processing steps^11,26,32^. Some studies have investigated the effects of various processing parameters on the density, dimensional accuracy, and surface finish of the part^8,10,11,16,18,20,22,25,27,34^. However, the understanding of the fundamental mechanisms is still limited. Previous studies have used *in situ* high-speed visible light imaging to observe the impact of droplets on the powder bed^22,35^. Particles were observed to agglomerate with the droplet impact to form spherical primitives^35^. An impact crater was observed to form around the primitive due to deformation of the powder bed from the moving primitive^35^. Additionally, particles around the droplet were observed to eject from the powder bed due to transfer of momentum from the binder droplet to the powder particles^22^. Postmortem observations of the printed layers also showed buried printed lines formed under the surface level of the powder bed from ejection of the powder particles adjacent to the printed line^22^. The previously reported *in situ* observations were recorded using a continuous jetting droplet print-heads which showed significantly different droplet formation mechanism compared to the droplet-on-demand (DoD) ink-jet print-heads commonly used on commercial binder jetting printers. Further, the optical imaging observations were limited to surface of the powder bed. Hence, no sub-surface information about powder bed dynamics and disruption has ever been obtained. Since the lines printed using DoD print-heads have been found to reside below the surface of the powder bed^22^, it is imperative to obtain sub-surface information to fully understand the physical processes involved in binder jetting.

Therefore, high-speed synchrotron hard X-ray imaging technique is used here to study the binder jetting AM processes of a variety of materials with high spatial (≈2 *μ*m) and temporal (≈5 *μ*s) resolutions. The X-ray images capture the highly dynamic phenomena above and inside the powder bed, revealing distinct behaviors of binder droplets, impact interaction between the droplet and the powder bed, and powder motion following the impact. The quantitative experimental data provides crucial insights into the binder jetting processes which will not only help reduce the defects and improve the quality of binder jetted parts, but also help develop and validate numerical models.

## Results and Discussion

### High-speed X-ray imaging

**Figure 1:**
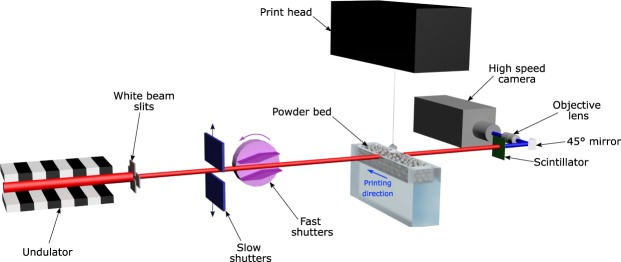
Schematic of the high-speed X-ray imaging experiments on binder jetting process at beam line 32-ID-B, Advanced Photon Source. A short-period undulator was used to generate a pseudo pink X-ray beam with first harmonic energy of 25.4 keV. The binder droplets impacted the powder bed from the top and the X-rays penetrated the sample from the side. The imaging detector was placed downstream, about 400 mm away from the sample. Shutters and slits were used to define the time window and size of the X-ray beam respectively.

The high-speed synchrotron X-ray imaging experiments were performed at beamline 32-ID-B, Advanced Photon Source (APS), Argonne National Laboratory. The schematic of the experimental setup is presented in Fig. 1. A previous experimental setup, used for studying laser powder fusion process^29,36,37^, was modified to accommodate a binder jetting printer in current experiments. More details of the experimental setup are provided in the “Methods” section.

### Binder droplet behavior

Comprehensive understanding the dynamics of generation and flight of binder droplets is essential for ensuring repeatability of droplets, accuracy in deposition, control of the droplet geometry and subsequently, quality of printed parts. The behavior of binder jetting print-heads was studied for continuous jetting printers before^33^, while in experiments presented here, the printer used a DoD ink-jet print-head for depositing the binder droplets on the powder beds. In DoD-type ink-jet printing, a droplet is generated using piezoelectric actuation with typical droplet velocities between 5 to 8 m/s^33^. DoD print-heads have been more popular in binder jetting processes due to their higher resolution, repeatability, and robust operations^33^.

**Figure 2:**
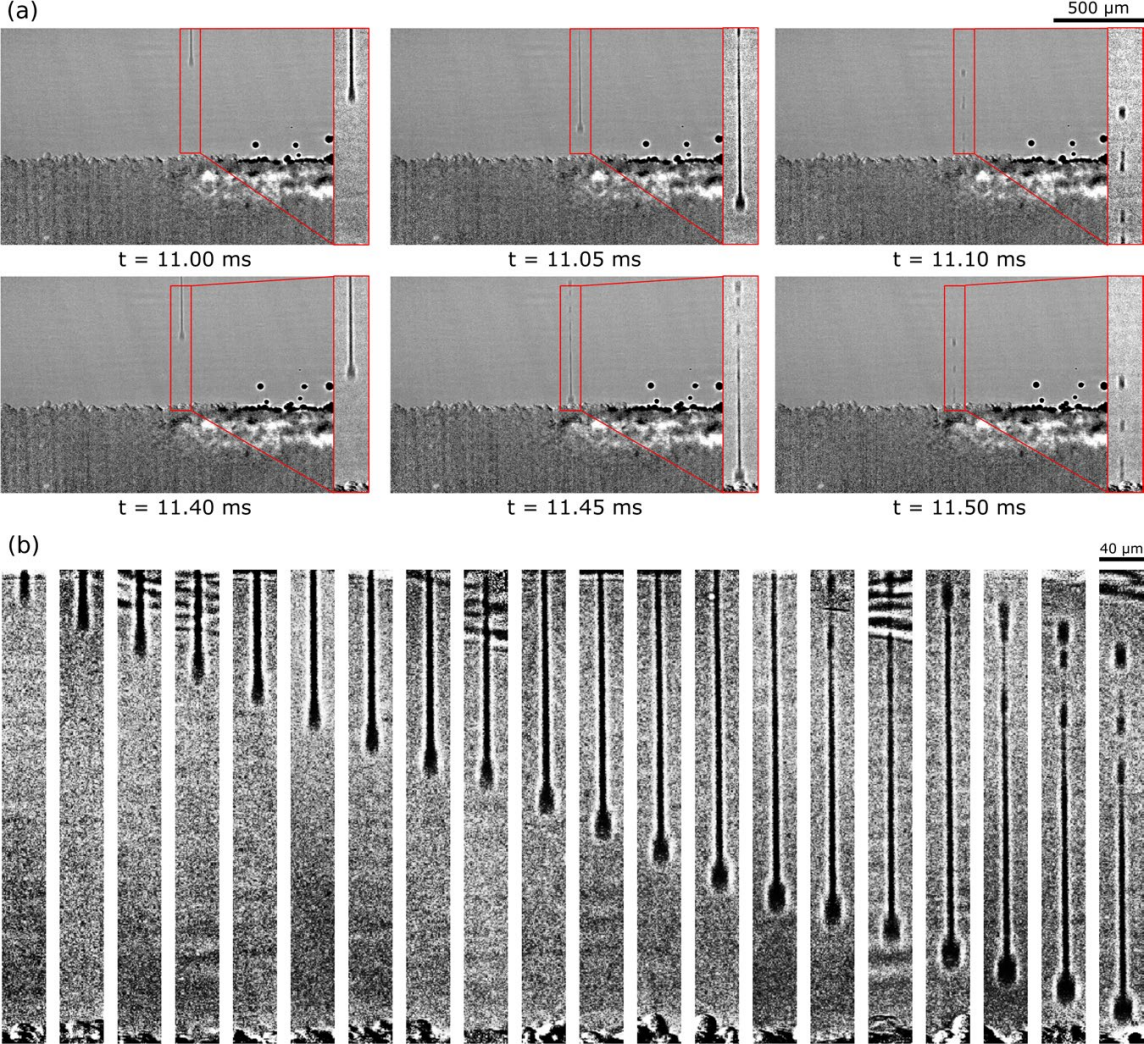
(**a**) Image sequence showing behavior of two consecutive binder droplets. Both droplets show jetted droplet shape with spherical head and long, narrow tail. Three to four satellite droplets are observed near the end of the tail. Nominal velocity of the droplet head was around 8 m/s. The temporal separation between two consecutive frames is 50 *μ*s. The size of the inset droplet figures is 120 *μ*m by 740 *μ*m. (**b**) Image sequence generated from different droplets showing the evolution in geometry of the droplet during flight. The head of the droplet becomes more spherical due to head pinch-off. The generation of satellites near the tails is clearly observed.

An image sequence for two consecutive droplets from a typical experiment is presented in Fig. 2(a). The droplets geometry displayed approximately spherical head followed by thin, long tail. The shape of the droplet was consistent with the droplet shapes reported earlier from ink-jet printers^38,39^. The diameter of the long tail was 12 ± 4 *μ*m. For each droplet, three to five satellite droplets were observed at the end of the tail. The experiments were repeated three times and total 117 droplets were analyzed. All measurements are reported with 95% confidence interval. The separation between the droplets was measured between the droplet head positions in the frame just before they impacted the powder bed. The measured separation between consecutive droplets was 49.34 ± 0.62 *μ*m which was very close to the separation set in the printer operation software. The measured velocity of the droplet head was 7.74 ± 0.06 *ms*^−1^, slightly lower than the designated velocity of 8 *ms*^−1^. The observed velocity for the last satellite for each droplet was 6.30 ± 0.05 *ms*^−1^. The tail of the droplet was observed to break up near the end, forming smaller satellite droplets. The length of the droplet tail before breakup was 703.16 ± 7.08 *μ*m (measured from 38 droplet images). The satellite droplets were observed to drift away from the droplet head in the direction of the print-head motion. The separation between the impact points for the droplet head and the satellite droplet was 15.12 ± 0.55 *μ*m.

From the binder fluid properties and the droplet geometry, the relevant dimensionless numbers were calculated as: Reynold’s number (Re) = 40.87, Weber number (We) = 54.90, Froude number (Fr) = 1.86 × 10^5^, and Ohnesorge number (Oh) = 0.18. Note that the droplet head diameter of 35 *μ*m was used as the droplet size in these calculations. Since the inverse of Froude number (1/Fr = 5.35 × 10^−6^) was significantly smaller than 1, the effect of gravity on the droplet behavior was negligible. The jetting behavior of the binder was observed to be dependent on the inverse of Oh, and the binders with Z = 1/Oh between 4 to 14 were observed to show good jettability^40^. The binder used in this study possesses good jettability with Z = 5.19 which is consistent with the bounds^40^. The droplet head shape changed from cylindrical to spherical as it traveled further as shown in Fig. 2(b). The evolution of the droplet head causes a neck region to form between the head and tail. This neck region can cause pinch-off thus separating the head and the tail regions^38^. In current experiments, the droplet impacted the powder bed before the head pinch-off occurred. The filament breakup at the back end of the droplet tail was caused by the Plateau-Rayleigh instability in the liquid stream^38,39^. A simple criterion for filament breakup was proposed by Hoath *et al*. where the critical aspect ratio (length/radius) for the filament breakup was given by $l/R=\sqrt{2}\alpha Oh$^39^ where *α* was determined to be around 33 based on previous experiments. For the current binder, the critical aspect ratio was calculated to be 8.45. From the experiments, the aspect ratio of the filament just before the breakup was 117.0 ± 10.4. Hence, the breakup of filaments into satellite droplets was consistent with the previously reported literature^38,39^. Further, the vertical speed of satellite droplets was slower than the droplet head, which is another evidence that the satellite droplets were formed by the filament instability. The splashing parameter *k*_*d*_ = *We*^0.5^*Re*^0.25^ was defined to assess if the droplet splashed into several smaller droplets upon impact^41^. Splashing was only observed for droplets with splashing parameter greater than 120^41^. For the current experiments, no splashing of the binder was observed in any experiments. The splashing parameter for the binder was 18.73; hence the lack of droplet splashing was again consistent with the reported critical parameter.

The drift between the droplet head and the satellite droplet was caused by the horizontal velocity of the print-head. As the droplet was being generated in the nozzle, the print-head was moving horizontally with the prescribed speed of approximately 0.12 m/s, which imposed a horizontal component of velocity on the droplet. The time separation between the impact of droplet head and the satellites was between 100 to 150 *μ*s (2–3 frames). The calculated horizontal displacement for the prescribed horizontal velocity is 12–18 *μ*m, which matches the experimentally observed values. From these observations, it is clear that the horizontal drift of the satellites will increase with horizontal velocity of the print-heads. Previously, the accuracy of the printed parts was observed to decrease with increasing printing speed^42^. The observations in our experiments suggest that the dimensional accuracy of the parts may decrease due to drift of satellite droplets with respect to the main droplet as the horizontal velocity of the print-heads is increased.

### Interaction depth and powder ejection

**Figure 3:**
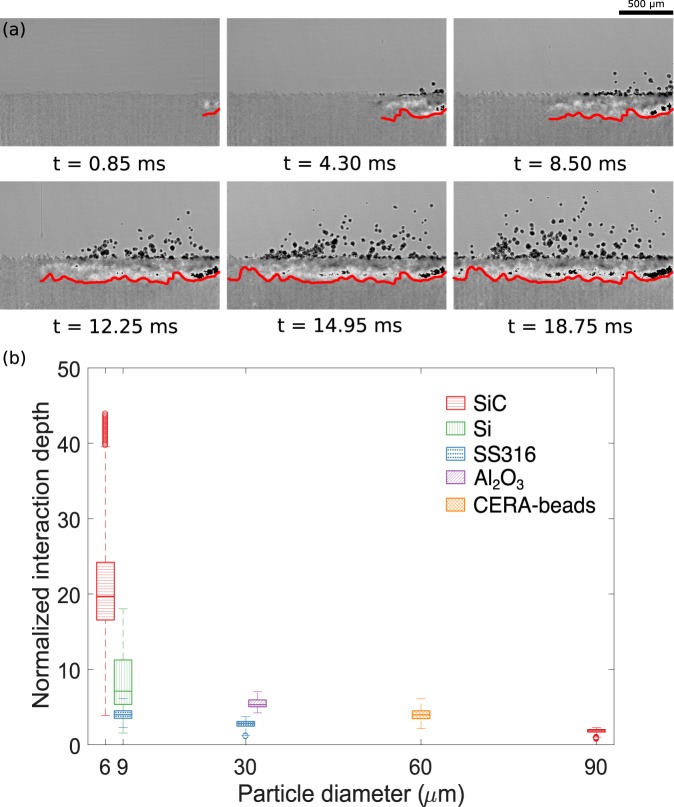
(**a**) Evolution of interaction depth for a binder jetting experiment (30 *μ*m stainless steel 316 powder). (**b**) Normalized interaction depths for powders. The interaction depths are normalized with the mean particle diameters (*d*_50_). For each particle type, the mean and standard deviation of the interaction depth measurement are plotted for all experiments.

The unique advantage of high-speed hard X-ray imaging is the ability to observe sub-surface behavior of the powder bed following the droplet impact. Six background corrected snapshots of the powder bed from a representative experiment are presented in Fig. 3(a). The disturbance in the powder bed due to impact of the droplets led to differences in the intensity in the image with respect to the image of the pristine powder bed. In this study, the depth of disturbance in the powder bed is termed as interaction depth. It should be noted that the observed interaction depth is different from the penetration depth for the binder, Here, the interaction depth depicts the changes in the powder bed due to movement and ejection of powder particles caused by the impact of the binder droplet and subsequent momentum transfer between the droplet and powder particles. Most of the binder droplet momentum was used to deform the powder bed, while a small percentage of the momentum (≈2%) contributed to ejection of the powder particles from the powder bed^43^. The interaction depth was mapped progressively as shown in Fig. 3(a). The mapped interaction depths were normalized with the mean particle diameters (*d*_50_ values). The normalized interaction depths for a variety of powders are plotted in Fig. 3(b). The mean size and shape of the powders studied here are listed in Table 1 in the Method section.

**Table 1:** Specifications of powders.

Material	Mean particle size (*d*_50_)	Particle shape	Particle density
Stainless steel 316	9 *μm* (*d*_10_ = 5 *μ*m, *d*_90_ = 22 *μ*m)	spherical	8000 kg/*m*^3^
	30 *μm* (*d*_10_ = 22 *μ*m, *d*_90_ = 53 *μ*m)	spherical	
Silicon carbide	6 *μm*	irregular	3210 kg/*m*^3^
	90 *μm*	irregular	
Aluminum oxide	32 *μm* (*d*_10_ = 14 *μ*m, *d*_90_ = 62 *μ*m)	irregular	3950 kg/*m*^3^
Silicon	9 *μm*	spherical	2320 kg/*m*^3^
CERA beads	60 *μm*	irregular	1690 kg/*m*^3^

From previous studies, impacts of fluid droplets on granular beds were observed to result in crater geometries^41,43,44^. The interaction depth values for large spherical free flowing particles were close to the analytical crater depths; while those for smaller particles with poor flowability were significantly different from the analytical crater depths (Supplementary material). Hence, impact cratering is believed to be the primary source of the disturbance in the powder bed for larger free flowing particles. For smaller poorly flowing particles, the disturbance was much deeper than the crater depth, which is speculated to be the result of the powder bed compression following droplet impact. As the particle size decreases, the cohesive forces between two particles were observed to increase^45^. Thus, the momentum from the droplet impact was transfered deeper for smaller particle sizes, leading to the increase in the normalized interaction depth. For similarly sized particles, the interaction depth was higher for irregular particles as compared to the spherical particles (Silicon vs. SS316 at 9 *μ*m and *Al*_2_*O*_3_ vs. SS316 around 30 *μ*m). Higher interaction depth for irregular particles may be attributed to better packing amongst the particles due to geometrical interlocking^46,47^. Further, the standard deviations for the interaction depths for irregular particles were higher than those for spherical particles. The uniformity of the powder bed (both packing fraction and powder surface roughness) was observed to decrease with increasing cohesiveness or decreased flowability of the powder with irregular particles^45^. Since the momentum transfer between the droplet and the powder particles depends strongly on the local packing of the powder bed^41^, the interaction depth changed significantly along the length of the bed for irregular particles, giving high standard deviation values.

**Figure 4:**
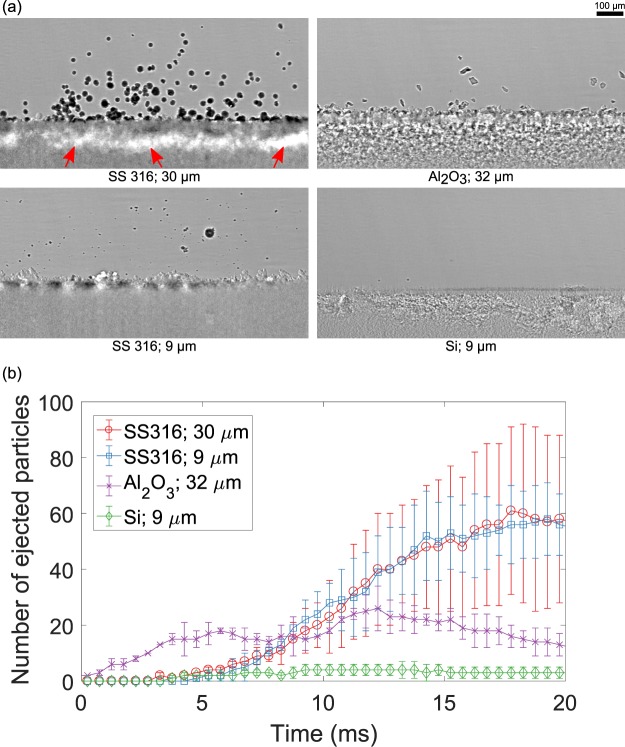
(**a**) Snapshots of high-speed x-ray videos from representative experiments at t = 18.8 ms showing ejection behavior of different powders. Red arrows indicate the depletion zone left in the powder bed. (**b**) Number of ejected particles as a function of printing time.

The ejection behavior of powder particles following the binder droplet impact was also dependent on size and morphology of the powder particles. High-speed frames from representative experiments for four different powders are presented in Fig. 4(a). The number of ejected particles for each powder is plotted in Fig. 4(b) as a function of printing time. The error bars reflect the standard deviation of the measurements from repeated experiments. The number of ejected particles was similar for both stainless steel 316 powders. Thus, the volume of ejected particles was significantly higher for the larger powder. Two mechanisms govern the ejection behavior of particles: since larger particles have better flowability, they are easier to displace from the powder bed. On the other hand, due to larger mass, each ejected particle accounts for more momentum for larger particle sizes. Interplay between two mechanisms resulted in both smaller and larger stainless steel powders having similar number of ejected particles. The number of ejected particles was comparatively smaller for *Al*_2_*O*_3_ and silicon powders. For similarly sized particles, the number of ejected particles was higher for spherical powders as compared to irregular particles. Irregular particles were observed to have poor flowability due to mechanical interlocking^46,47^, which may explain the smaller number of ejected particles for irregular particles. Further, the number of airborne particles decreased near the end of recording for *Al*_2_*O*_3_ powder due to the particles falling back to the bed. On the other hand, the number of ejected particles increased with time for stainless steel powders with ejected particles staying airborne for longer. This indicated that the initial ejection velocity was higher for stainless steel particles. For powders that showed large volume of ejected particles (SS316, 30 *μ*m), a sub-surface depletion zone was formed under a thin layer of powder as shown in Fig. 4(a). The depth of depletion layer for SS316: 30 *μ*m powder was 56 ± 12 *μ*m.

The study of interaction depth and powder ejection provides significant implications on the possible mechanisms for the defects formation in binder jetting processes. First, deep interaction depths due to particle motion can potentially create sub-surface pores in printed parts as the binder penetration time is typically much larger than the time required for particle motion in the bed, thus allowing binder to permeate in a disturbed powder bed. Second, if a large number of particles are ejected, sub-surface depletion zones form in powder beds, which will generate defects in parts through various mechanisms. If the depletion zone is not refilled in subsequent layers, it will result in large pores in the final part. Spreading of subsequent powder layers may refill or collapse the depletion zone. This will lead to variation in the local thickness of the powder bed causing significant dimensional inaccuracies. Third, the ejected powder particles settle in different positions on the powder bed compared to their initial positions. The settled powder particles will affect the spreading of the subsequent powder layer negatively and increase the chance of getting a part with inaccurate dimension and large roughness.

### Agglomeration behavior

**Figure 5:**
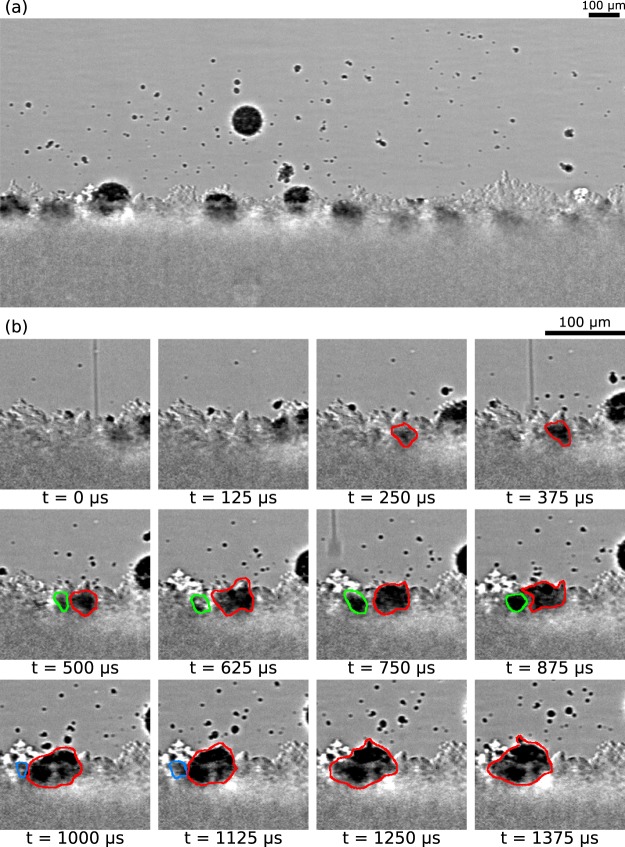
(**a**) A representative frame from stainless steel 9 *μ*m powder experiment showing the formed agglomerates both in air and on the powder bed. (**b**) Frame sequence showing the formation of an agglomerate. The agglomerate was formed by coalescence of three consecutive binder droplets. The red, green, blue outlines show the primitives formed from first, second, and third droplet respectively. The consecutive primitives merge to form the large agglomerate.

A distinct agglomeration behavior was observed for powders with smaller particle sizes (typically < 10 *μ*m). Several large agglomerates formed in 9 *μ*m SS316 powder during the printing process are presented in Fig. 5(a). For 9 *μ*m SS316 powder, the agglomerate diameter was 95.6 ± 10.9 *μ*m (24 measurements). The mechanism of agglomerate formation is presented in Fig. 5(b). Typically, each agglomerate was formed by the merging of three consecutive droplets. The first droplet formed a primitive in front of the droplet which merged with the primitives from the next two droplets. After merging of three primitives, the powder in the vicinity of the next droplet was sufficiently denuded such that the next primitive was formed only in front of the droplet location at a certain distance away, initiating a new agglomerate. The diameters of the agglomerates were between 1.8 to 3.8 times the diameter of the binder droplets, which is consistent with the merging of three droplets to form the agglomerate. Typically, the ejected agglomerates showed smaller diameters, where free agglomerates attained a spherical shape because of the surface tension of the binder. Larger diameters were observed for hemispherical agglomerates adhered to the powder bed, where droplet spreading following impact increased the diameter.

Agglomeration of powder particles following a liquid droplet impact has been studied for wet granulation techniques^48–51^. For a given binder deposition rate, the agglomeration behavior was observed to change from drop controlled agglomeration (each droplet forming one agglomerate) to mechanical dispersion controlled agglomeration (droplet coalascencing on the powder surface causing larger agglomerates) as the penetration time was increased for the binder droplets^48^. The penetration time for binder droplets deposited on loosely packed powder beds increases as the particle size is decreased as shown in the supplementary material. The penetration time determines how long the binder stays near the top of the powder bed. For smaller particle sizes, the binder droplet stays near the top of the bed for longer and merges with the next binder droplet, thus forming a larger agglomerate. For larger particles, the binder wicks into the powder bed faster and hence does not form large agglomerates. Hence, the agglomeration regime changed from the drop controlled agglomeration to dispersion controlled agglomeration as the particle size was decreased. These predictions match the experimental observations where smaller particles showed coalescence of droplets to form larger agglomerates.

The observed agglomeration behavior may lead to various defects in the printed part. In previous studies, the powder particles were observed to segregate towards the surface of the agglomerate, thus leaving a core composed entirely of binder^22,35^. When the binder is burned away, the agglomerate will leave large pores, and dimensional inaccuracy occurs if those pores collapse. The large agglomerates will also interfere in uniform spreading of the next powder layer.

## Conclusions

In the present contribution, some important physical processes involved in binder jetting AM were investigated *in-situ* using high-speed synchrotron X-ray imaging. Owing to the superior penetration power of hard x-rays, the high-speed imaging technique at the APS allows the capture of the dynamic binder-powder interaction inside the powder bed with high spatial and temporal resolutions. Revealed in the experiments, the binder droplets contained an elongated shape with round head, narrow tail, and several trailing satellite droplets. The satellite droplets showed significant drift with respect to the main droplet, which may introduce dimensional errors in the printed parts. The disturbance in the powder bed induced by the binder impact both shifted the particles under the droplet and ejected the particles from the bed. The depth of disturbance in the powder bed was defined as interaction depth. The normalized interaction depth was observed to decrease with increasing particle size because of the reduced cohesive forces between the particles. Further, normalized interaction depth was higher for irregular particles as compared to the spherical particles because of higher mechanical interlocking between irregular particles. It was postulated that impact cratering and powder bed compression were the major mechanisms for bed disturbance in spherical free flowing and irregular cohesive particles, respectively. The number of ejected particles was higher for spherical free flowing powders as compared to the irregular powders. The large volume of ejected particles for large spherical powder left a depleted zone in the wake of the binder. Both sub-surface motion and ejection of particles will potentially increase porosity in the printed parts. For particles with diameter less than 10 *μ*m, three consecutive binder droplets were observed to coalesce to form large spherical agglomerates. These agglomerates are likely to interfere with spreading of the next powder layer to generate defects. Further, the agglomerates will also lead to defects as all the particles were segregated to the surface of the agglomerate.

Binder jetting offers many advantages over other AM techniques for printing specific materials, but its full potential can only be realized with a comprehensive understanding of the sophisticated relationship between the feed stock characteristics, processing conditions, and the part quality. So far, binder jetting technique has been successfully used to print many high quality parts from various materials. The study shown here represents an early, yet successful, attempt to probe the mechanisms that are responsible for the formation of different defects in binder jetting AM parts. An extensive application of this high-resolution *in situ* X-ray characterization technique will undoubtedly facilitate not only the improvement of binder jetting printers for reliably building defect-free parts, but also the development of high-fidelity numerical models for optimizing the process parameters for manufacturing parts composed of different materials and geometries.

## Methods

### Materials

Five different materials and seven different powder material-particle size combinations were studied. The details of the powders are presented in Table 1. These combinations were chosen to study effects of material properties, mean particle size and morphology on the binder jetting process. The particle size distribution (*d*_10_ and *d*_90_ values) are provided for some powders. All powders studied here are commonly used in binder jetting AM. The mean particle diameter represents the equivalent diameter of the spherical particle with equal volume.

### Binder jetting setup

For the *in situ* high-speed X-ray imaging experiments, the powder bed specimens were prepared manually by pouring the powder particles in a polycarbonate holder with channel width of 500 *μm* and channel depth of 1 mm followed by scraping of the top to obtain a flat surface.

The binder jetting experiments were performed on a commercially available X1-Lab printer (ExOne, North Huntingdon, PA). In each experiment, a single line was printed on the powder bed in direction perpendicular to the X-ray propagation direction. Only one nozzle was used to deposit a single binder droplet (volume = 30 pl) at a time. The separation between consecutive droplets was set at 50 *μm* and the horizontal printing speed was set to 120 mm/s. Print head to powder bed distance was approximately 2 mm. In studies reported by others, distance between the nozzle and powder bed was observed to have negligible effect on the behavior of powder bed under printing conditions^22^. A proprietary aqueous binder provided by ExOne was used in the experiments. Viscosity, surface tension, and density of the binder was 6.85 × 10^−3^ Pa.s, 40.8 × 10^−3^ N/m and 1000 kg/*m*^3^ respectively.

### High-speed X-ray imaging

The high-speed X-ray imaging experiments were performed at the 32-ID-B beamline, Advanced Photon Source, Argonne National Laboratory. Previously, this technique has been used to study the laser powder bed fusion additive manufacturing process^29,36,37^. Polychromatic X-rays were generated using a short-period (18 mm) undulator with the gap set at 17 mm. The first harmonic energy of the X-rays was centered at 25.4 keV (*λ* = 0.488 Å). White beam slits were used to set the X-ray beam window to approximately 2.1 × 2.1 *mm*^2^ which provided the integrated photon flux of ≈2 × 10^14^ photons/s. A set of slow and fast shutters were utilized to define a small time window when X-rays were shining on the sample. An array of delay generators (DG535, Stanford Research Systems, Sunnyvale, CA USA) was used to synchronize the binder jetting event, X-ray shuttering, and high speed camera trigger. The X-ray beam was allowed to pass through the sample and was subsequently converted to a visible light signal using a single crystal *Lu*_3_*Al*_5_*O*_12_:*Ce* scintillator. The visible light images were recorded using a high-speed camera (Photron SA-Z, Photron Limited, Tokyo, Japan). The image recording speed was 20,000 frames/s. The exposure time for each frame was 5 *μs*. The resolution of the imaging system was 1.98 *μm*/*px* and the frame size was 1024 × 1024 pixels.

In a typical experiment, a ‘start’ signal (t = 0 s) was sent from the control software to the printer. The machine required approximately 52.5 s to prime the print-head and clean the nozzles. The actual printing process started at t = 52.5 s. The trigger signal for the camera was sent at t = 55.1 s. The print-head nozzle entered and exited the X-ray field-of-view at approximately t = 55.103 s and 55.118 s respectively.

The recorded images were normalized with the static image (first frame) of the image sequence to improve the contrast such that small changes in the powder bed were clearly visualized. The brightness and contrast of the normalized images were further adjusted. All image processing steps were performed using an open source image processing software (ImageJ^52^).

## Supplementary information

Real time observation of binder jetting printing process using high-speed X-ray imaging: Supplementary material

## Data Availability

The raw and processed data are available from the corresponding authors on reasonable request.
