# Supplementary material for: Real time observation of binder jetting printing process using high-speed X-ray imaging
Source: Sci Rep. 2019 Feb 21;9:2499. doi: 10.1038/s41598-019-38862-7 (PMC6385361; doi:10.1038/s41598-019-38862-7)
Supplement: Supplementary file 1 — Real time observation of binder jetting printing process using high-speed X-ray imaging: Supplementary material [file 41598_2019_38862_MOESM1_ESM.pdf]

Real time observation of binder jetting printing process using high-speed X-ray imaging: Supplementary material

Niranjan D. Parab[1], John E. Barnes[*2], Cang Zhao[1], Ross W. Cunningham[3], Kamel Fezzaa[1], Anthony D. Rollett[†3], and Tao Sun[‡1]

[1]X-ray Science Division, Advanced Photon Source, Argonne National Laboratory, Argonne, IL, 60439, USA
[2]The Barnes Group Advisors, Pittsburgh, PA, 15143, USA
[3]Department of Materials Science and Engineering, Carnegie Mellon University, Pittsburgh, PA, 15213, USA

ABSTRACT

This documents provides additional data and information for the paper titled 'Real time observation of binder jetting printing process using high-speed X-ray imaging'.

Dependence of porosity on particle size

The dependence of porosity in loosely packed powder beds on size of the fine particles was reported earlier[1]. A logarithmic equation was fitted to the porosity values measured from experiments and computer simulations. The fitting equation is:

$$\varepsilon = -0.0975 log(D_p) + 0.8391 \tag{1}$$

where ε is the porosity and D_p is the particle diameter. Experimental porosity values and the fitted curve are presented in Figure 1.

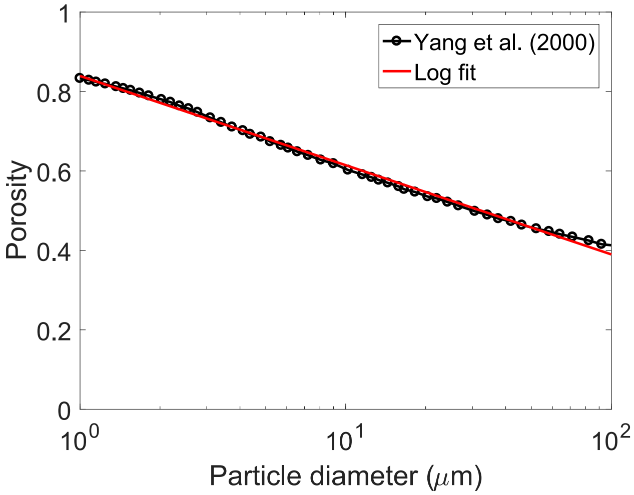

Figure 1. Size dependence for porosity in packing of fine particles[1]

For poly-disperse particle distribution, the porosity values were observed to be slightly lower than that for mono-disperse distributions. However, the difference between the porosities was small ($\approx$ 10%)[2,3]. Hence, in subsequent discussion, the particle size distributions are assumed to be mono-disperse with particle size represented by the average particle diameter (d_{50})

*John@thebarnes.group
†rollett@andrew.cmu.edu
‡taosun@aps.anl.gov

Crater dimensions and interaction depth measurements

From previous studies, impact of fluid droplet on granular beds were observed to result in crater geometries[4–6]. The radius of the crater was observed to follow equation 2[4].

$$R = \frac{1}{2} R_l \left(\frac{\rho_{bulk}}{\rho_{binder}} \right) We^{0.25} \quad (2)$$

where R_l was the radius of the droplet, ρ_{bulk} was the bulk density of the powder bed, ρ_{binder} was the density of the binder, and We was the Weber number. The ratio of the crater depth to crater diameter was observed to be around 0.2 for loosely packed granular beds[7,8]. Considering the radius of the droplet to be the radius of the head (R_l = 18 μm) and density of stainless steel and silicon carbide equal to 8000 kg/m^3 and 3210 kg/m^3 respectively, the crater depths are calculated and plotted in Figure 2.

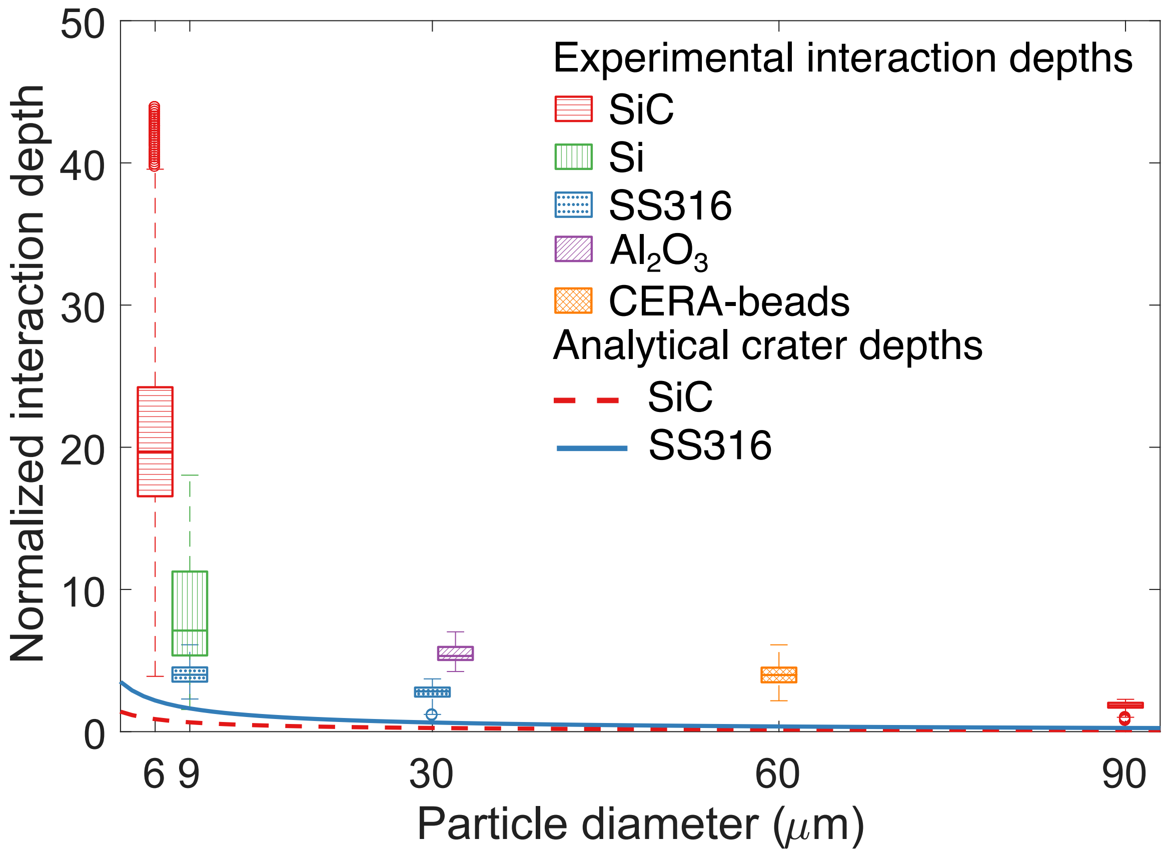

Figure 2

The interaction depth values for large spherical, free flowing particles were close to the analytical crater depths; while those for smaller particles with poor flowability were significantly different from the analytical crater depths. Hence, impact cratering may be the primary source of the disturbance in the powder bed for larger, free flowing particles. A large depletion zone was also observed for large, free flowing particles in wake of the binder, which further indicates that impact cratering is the major contribution to interaction depth.

Droplet penetration time and agglomeration behavior model

For binder droplet impacting loosely packed powder bed, the penetration time for the droplet is given by[9]:

$$t_{pen} = 1.35 \frac{V_0^{2/3}}{\varepsilon_{eff}^2 R_{eff}} \frac{\mu}{\sigma cos\theta} \quad (3)$$

where V_0 is the initial droplet volume, μ the liquid viscosity, σ the surface tension, θ the contact angle, ε_{eff} the effective powder bed porosity, and R_{eff} the effective pore radius. Using the Kozeny equation, the effective pore radius is given by[10]:

$$R_{eff} = \frac{D_p}{3} \frac{\varepsilon_{eff}}{1 - \varepsilon_{eff}} \tag{4}$$

where D_p is the particle diameter. The dependence of the penetration time for a given particle-binder material pair on the particle size based on the fitted porosity values is plotted in Figure 3.

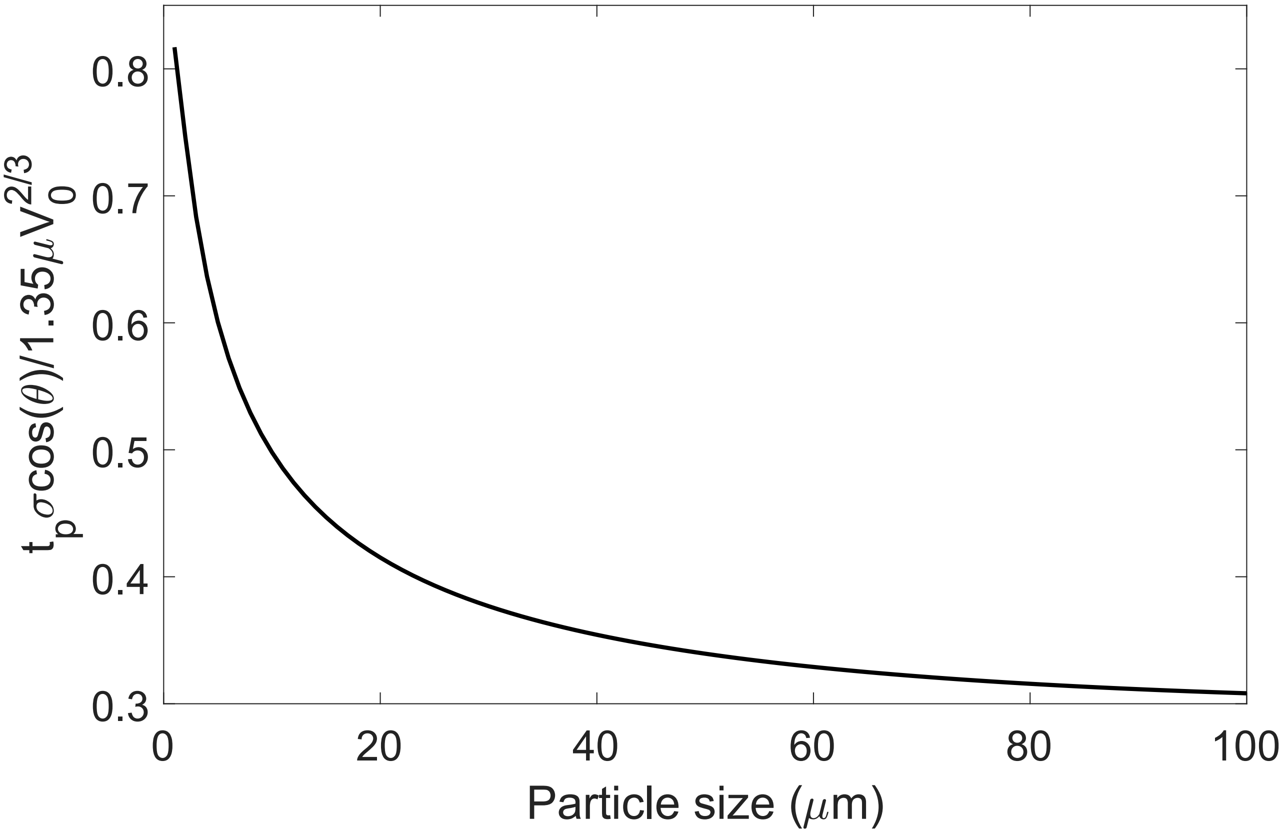

Figure 3. The penetration time for a given droplet increases with decreasing particle size thus changing the agglomeration behavior from drop controlled to dispersion controlled agglomeration

The penetration time is expected to increase as the particle size is decreased. For a given particle-binder material system with constant binder deposition rates, the agglomeration behavior was observed to change from drop controlled agglomeration (each droplet forming one agglomerate) to mechanical dispersion controlled agglomeration (droplet coalescing on the powder surface causing larger agglomerates) as the penetration time increased. From the experiments, for particle diameters smaller than 10 μm, multiple binder droplets were observed to coalesce to form larger agglomerates thus showing dispersion controlled agglomeration, which matches the predicted agglomeration behavior.

References

1. Yang, R. Y., Zou, R. P. & Yu, A. B. Computer simulation of the packing of fine particles. *Phys. Rev. E* **62**, 3900–3908 (2000).
2. Schulze, S., Nikrityuk, P. A. & Meyer, B. Porosity Distribution in Monodisperse and Polydisperse Fixed Beds and Its Impact on the Fluid Flow. *Part. Sci. Technol.* **33**, 23–33, DOI: 10.1080/02726351.2014.923960 (2015).
3. Wang, J.-P., François, B. & Lambert, P. Equations for hydraulic conductivity estimation from particle size distribution: A dimensional analysis. *Water Resour. Res.* **53**, 8127–8134, DOI: 10.1002/2017WR020888.

4. Katsuragi, H. Length and time scales of a liquid drop impact and penetration into a granular layer. *J. Fluid Mech.* **675**, 552–573, DOI: 10.1017/jfm.2011.31 (2011).

5. Long, E. J. *et al.* Experimental investigation into the impact of a liquid droplet onto a granular bed using three-dimensional, time-resolved, particle tracking. *Phys. Rev. E* **89**, 32201, DOI: 10.1103/PhysRevE.89.032201 (2014).

6. Nefzaoui, E. & Skurtys, O. Impact of a liquid drop on a granular medium: Inertia, viscosity and surface tension effects on the drop deformation. *Exp. Therm. Fluid Sci.* **41**, 43–50, DOI: https://doi.org/10.1016/j.expthermflusci.2012.03.007 (2012).

7. de Jong, R., Zhao, S.-C. & van der Meer, D. Crater formation during raindrop impact on sand. *Phys. Rev. E* **95**, 42901, DOI: 10.1103/PhysRevE.95.042901 (2017).

8. Zhao, R., Zhang, Q., Tjugito, H. & Cheng, X. Granular impact cratering by liquid drops: Understanding raindrop imprints through an analogy to asteroid strikes. *Proc. Natl. Acad. Sci.* **112**, 342–347, DOI: 10.1073/pnas.1419271112 (2015).

9. Hapgood, K. P., Litster, J. D. & Smith, R. Nucleation regime map for liquid bound granules. *AIChE J.* **49**, 350–361, DOI: 10.1002/aic.690490207.

10. Lee, A. & Sojka, P. Drop impact and agglomeration under static powder bed conditions. *AIChE J.* **58**, 79–86, DOI: 10.1002/aic.12575.
